# Supplementary figures and images for: Complement Decay-Accelerating Factor is a modulator of influenza A virus lung immunopathology
Source: PLoS Pathog. 2021 Jul 1;17(7):e1009381. doi: 10.1371/journal.ppat.1009381 (PMC8248730; doi:10.1371/journal.ppat.1009381)

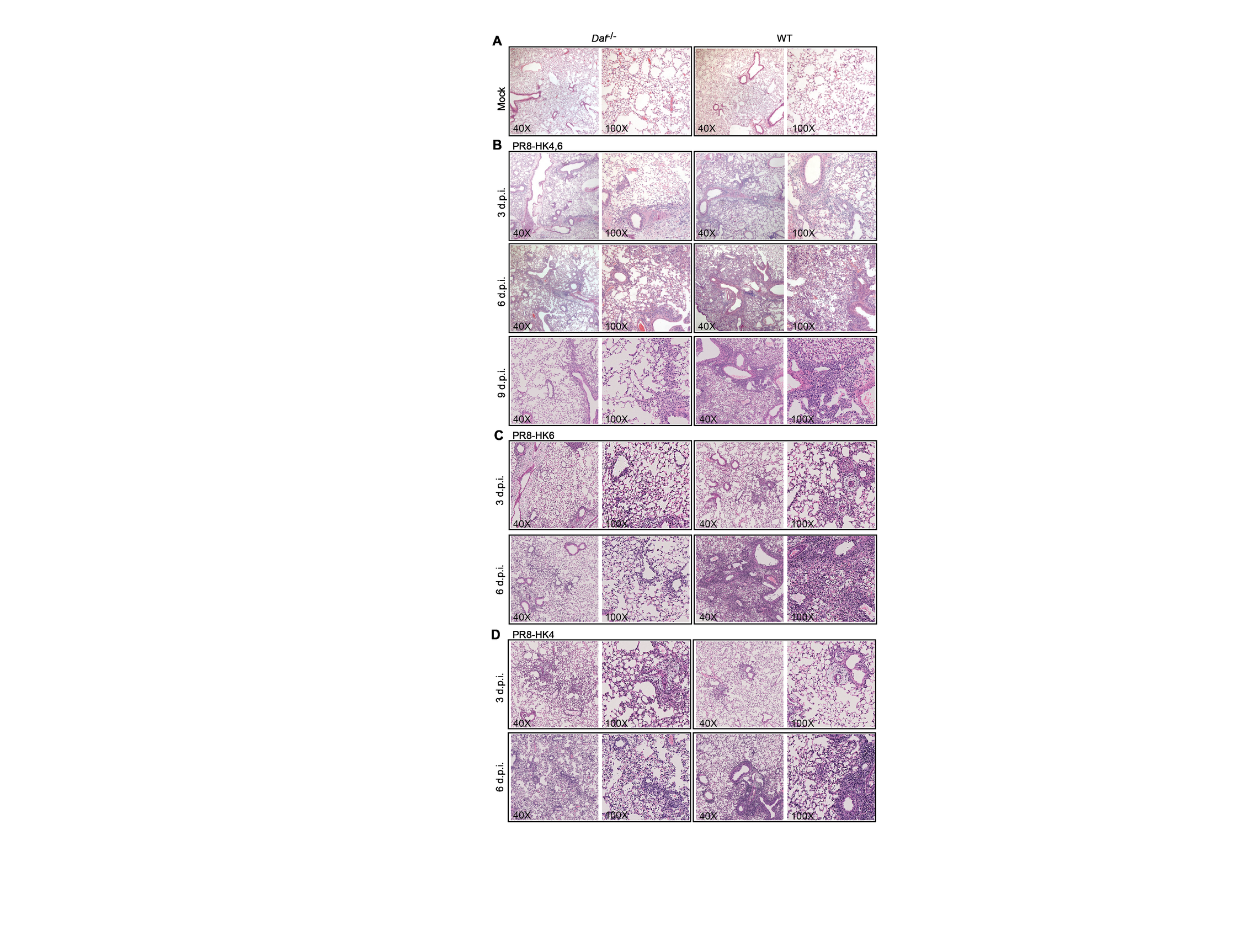

Supplement: S1 Fig — C57BL/6J WT and Daf-/- mice were mock infected (A) or infected with 1000 PFU of PR8-HK4,6 (B), 20 PFU of PR8-HK6 (C) and 100 PFU of PR8-HK4 (D). Samples were collected at the indicated time points. (TIF) [file ppat.1009381.s001.tif]

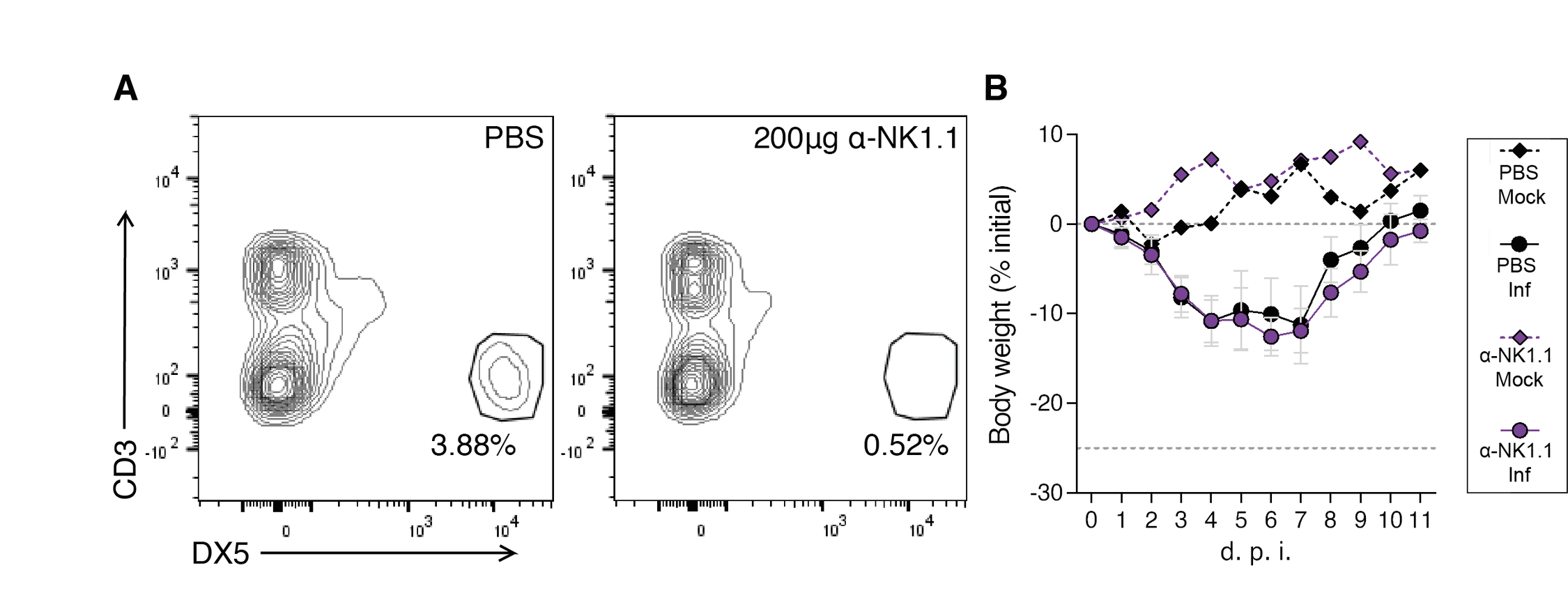

Supplement: S2 Fig — A: Representative flow cytometry detection of NK cells (gated in CD45+ population) in C57BL/6J WT 72 hours after depletion via intraperitoneal (IP) injection of α-NK1.1. B: Bodyweight loss of C57BL/6J WT mice infected with 100 PFU of A/X-31 (PR8-HK4,6) and depleted of NK cells by IP injection of α-NK1.1 every 72 hours, starting 72 hours before infection (Inf n = 5 and mock n = 1 per group). Results are expressed as mean±sd. Statistical analysis detailed in materials and methods. (TIF) [file ppat.1009381.s002.tif]

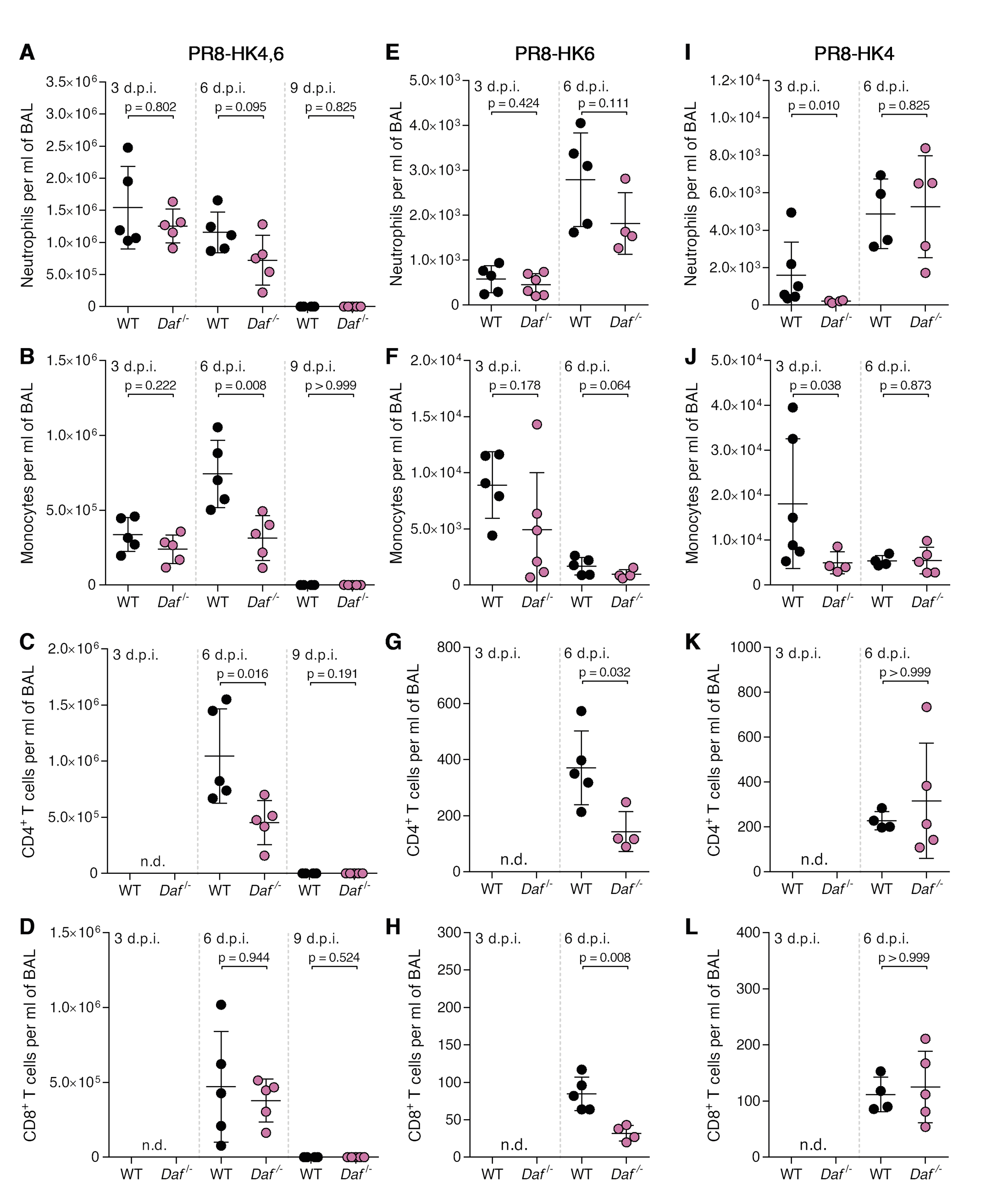

Supplement: S3 Fig — A, B, C, D: Analysis of neutrophils (A) monocytes (B) CD4+ T cells (C) and CD8+ T (D) cells levels in BALs of WT or Daf-/- mice infected with 1000 PFU A/X-31 (PR8-HK4,6). Samples were collected at 3 d.p.i. (n = 4 per group) and 6 d.p.i. (n = 5 per group) and 9 d.p.i. (n = 5 per group). E, F, G, H: Analysis of neutrophils (E) monocytes (F) CD4+ T cells (G) and CD8+ T (H) cells levels in BALs of WT or Daf-/- mice infected with 1000 PFU PR8-HK6. Samples were collected at 3 d.p.i. (n = 5 and n = 6 for WT and Daf-/- respectively), and 6 d.p.i. (n = 5 per group). I, J, K, L: Analysis of neutrophils (I) monocytes (J) CD4+ T cells (K) and CD8+ T (L) cells levels in BALs of WT or Daf-/- mice infected with 1000 PFU PR8-HK4. Samples were collected at 3 d.p.i. (n = 6 and n = 5 for WT and Daf-/- respectively) and 6 d.p.i. (n = 4 and n = 5 for WT and Daf-/- respectively). Samples with non detectable cell levels are noted as n.d. (TIF) [file ppat.1009381.s003.tif]

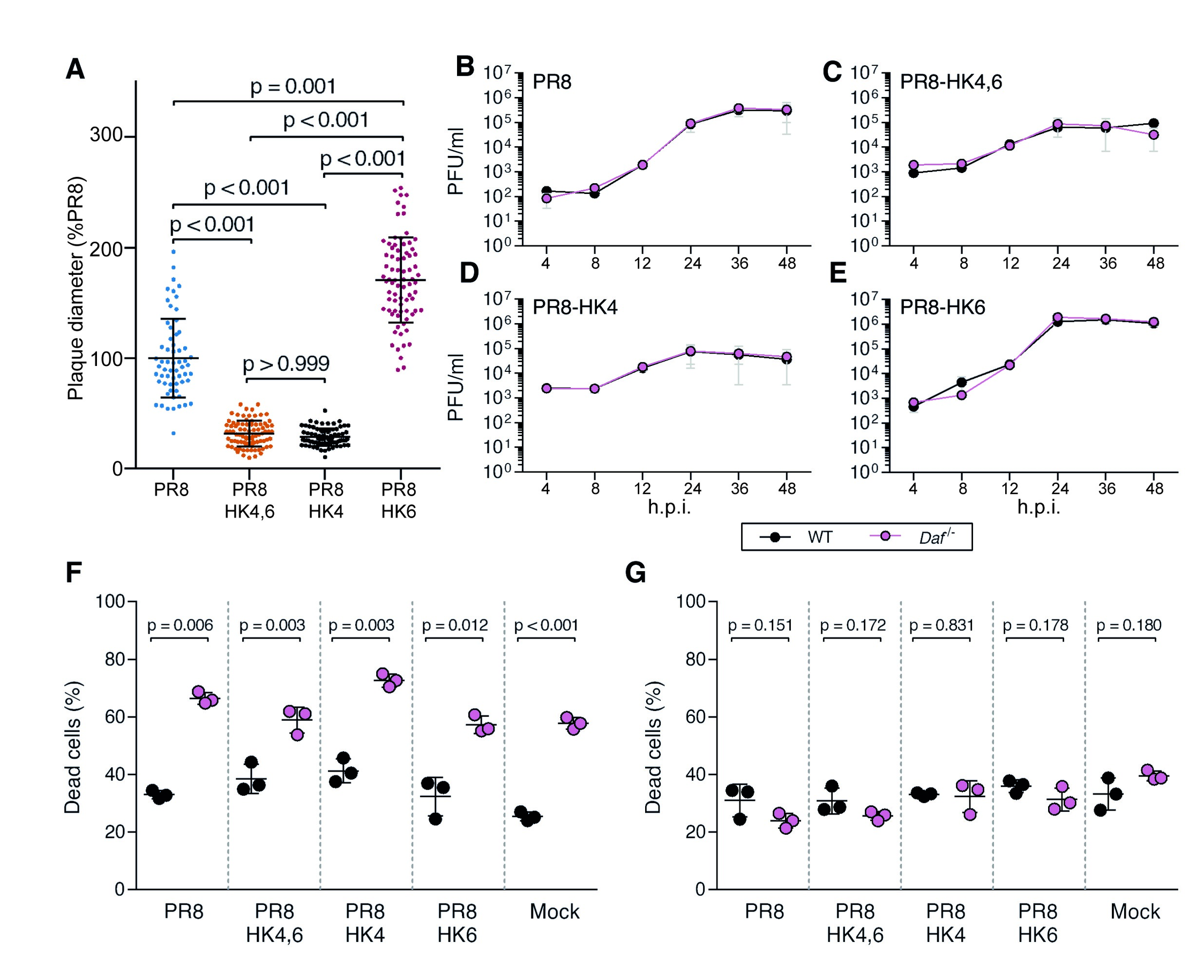

Supplement: S4 Fig — A: Measurement of viral plaques diameter after infection of MDCK cells monolayers. Data shown as mean±sd from two independent experiments, each corresponding to six independent infections for each virus. Each point represents an individual plaque. B-E: Replication kinetics of A/Puerto Rico/8/1934 (PR8) (B), A/X-31 (PR8-HK4,6) (C), PR8 containing the segment 4 of A/Hong Kong/1/68 (HK68) (PR8-HK4) (D) and PR8 containing the segment 6 of HK68 (PR8-HK6) (E) in mouse embryonic fibroblasts (MEFs) derived from C57BL/6J WT or Daf-/- mice at multiplicity of infection (MOI) = 0.005. Data shown as mean±SEM, from two independent experiments. Statistical analysis detailed in materials and methods. F, G: Cell death of primary lung cells derived from WT or Daf-/- mice infected or mock-infected with PR8, PR8-HK4,6, PR8-HK4 or PR8-HK6 and treated with serum (F) or heat-inactivated control (G). Results are expressed as mean±sd from three replicates from two independent experiments. Statistical analysis detailed in materials and methods. (TIF) [file ppat.1009381.s004.tif]

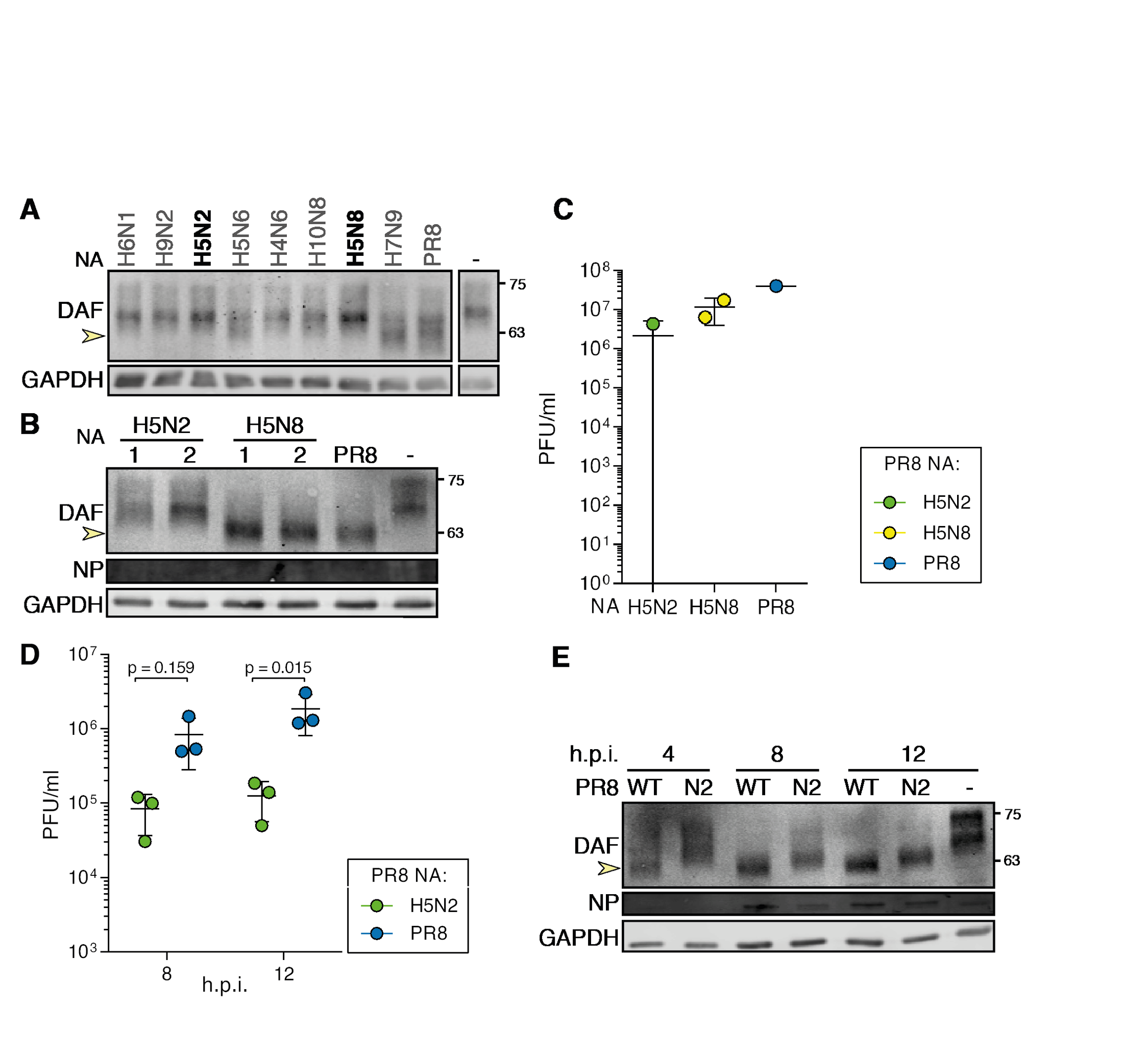

Supplement: S5 Fig — A: As showed in Fig 10I, HEK293T cells were transfected with eight different avian derived NAs. B: To produce reverse genetics (RG) reassortant A/Puerto Rico/8/34 (PR8) viruses, HEK293T cells were transfected with seven plasmids encoding segments 1–5, 7 and 8 from PR8, and the segment 6, which encodes NA, from the indicated viruses. C: After one round of amplification in embryonated chicken eggs, recovered viruses were titrated. D-E: A549 cells were infected with PR8 NA-H5N2 at multiplicity of infection (MOI) of 3 and samples collected at the indicated timepoints to titrate released virions (D) and analyze DAF cleavage by western blot (E) (D: pooled data from three independent experiments; E: representative blot from three independent experiments). Statistical analysis detailed in materials and methods. (TIF) [file ppat.1009381.s005.tif]

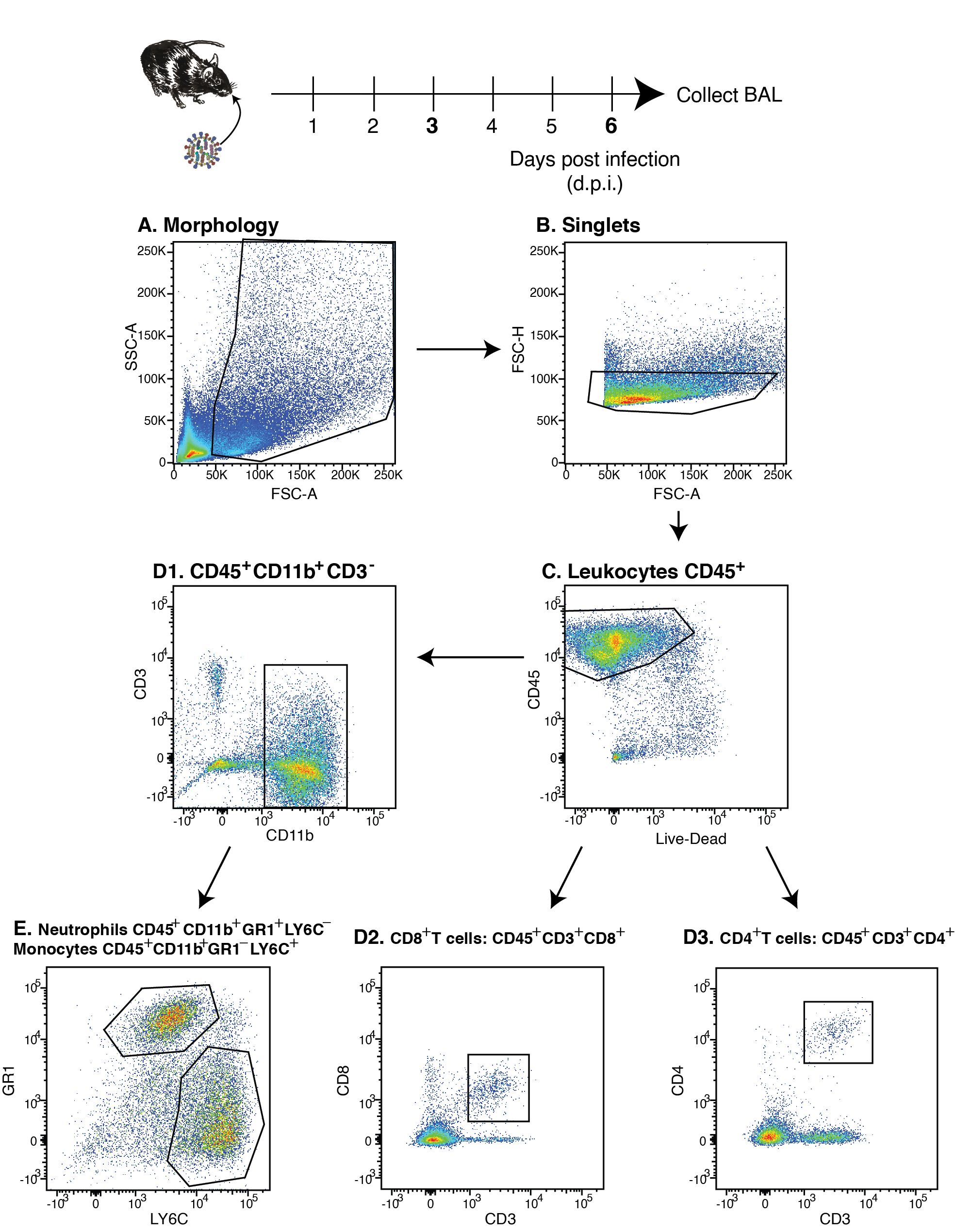

Supplement: S6 Fig — (TIF) [file ppat.1009381.s006.tif]

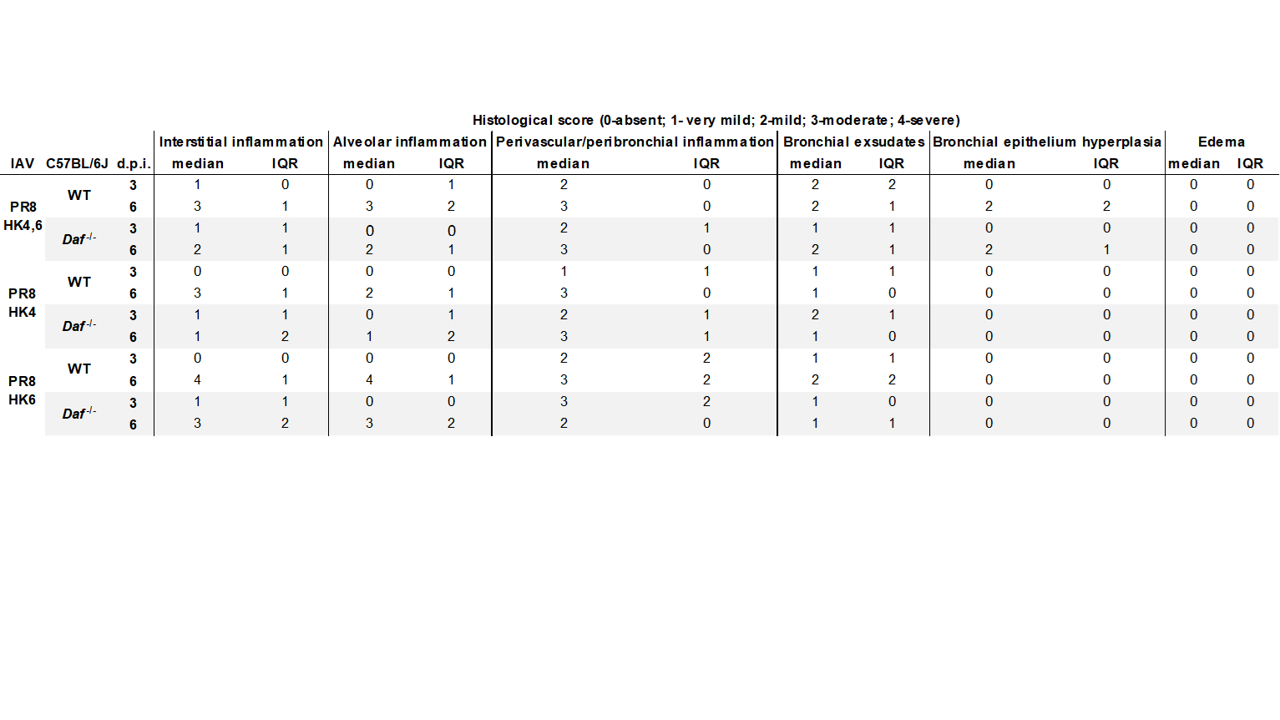

Supplement: S1 Table — (TIF) [file ppat.1009381.s007.tif]

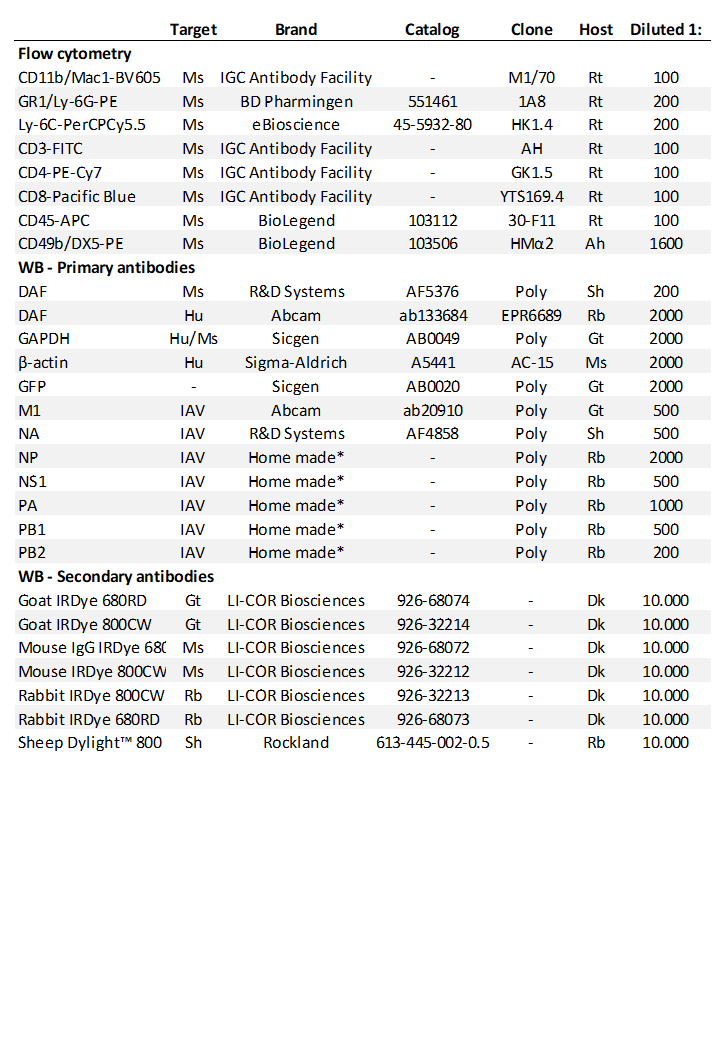

Supplement: S2 Table — (TIF) [file ppat.1009381.s008.tif]

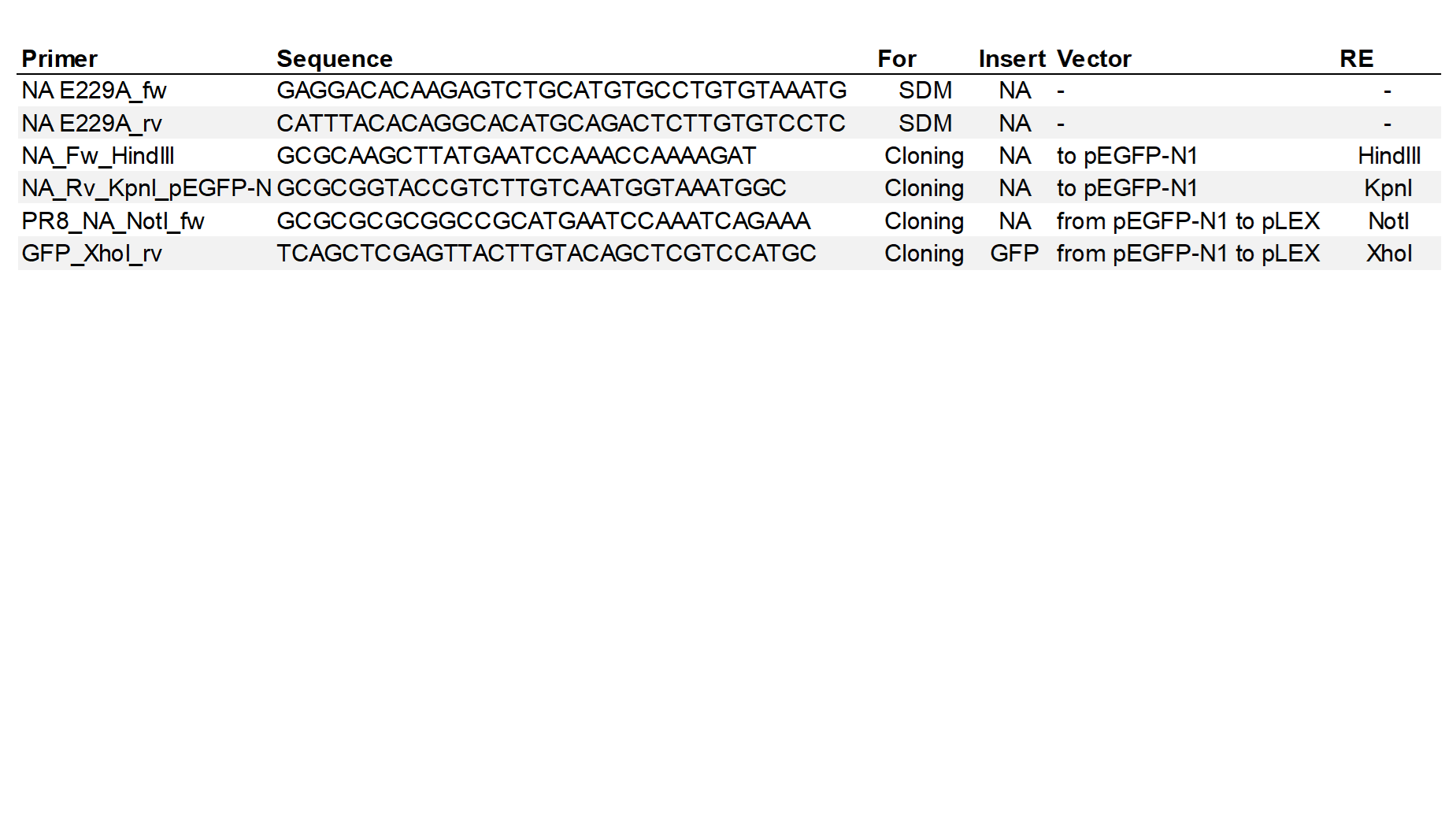

Supplement: S3 Table — (TIF) [file ppat.1009381.s009.tif]
