# Supplementary material for: Complement Decay-Accelerating Factor is a modulator of influenza A virus lung immunopathology
Source: PLoS Pathog. 2021 Jul 1;17(7):e1009381. doi: 10.1371/journal.ppat.1009381 (PMC8248730; doi:10.1371/journal.ppat.1009381)
Supplement: Attachment — Submitted filename: Answers to reviewers.docx [file ppat.1009381.s010.docx]

The authors thank this reviewer for taking the time to evaluate their manuscript and for contributing to improving it. We have answered each question below and we believe we have addressed most, if not all, this reviewers’ concerns.

Fig 2c – Can the authors describe what the end point in their survival experiment was? In the methods is appears a weight loss >25% was grounds for euthanasia. If this is the case, it is unclear how the WT mice which lost less weight compared to the *Daf^-/-^* mice have worse survival rates. Can the individual weight loss curves for each mouse be presented instead.

We are aware that as we plotted the mean values, when a mouse is euthanized for losing more than 25% of the initial bodyweight, the mean increases and, this means to be better explained in the text. We opted for the mean value, as given the number of mice used in each experiment, presenting individual weight loss curves for each mouse would not allow a clear understanding of the results. To clarify this issue, we have corrected the text as follows:

“Animals that presented a body weight loss of more than 25% were closely monitored. To comply with best animal welfare practices, animals that maintained a reduction of more than 25% for 24h were sacrificed. However, animals that regained enough weight to be above the -25% threshold after those same 24h were allowed to recover.”

3) You mention for Fig 1D that the survival is 25% and it is more like 30%

We acknowledge that a better explanation is required, and have corrected the text as follows:

“Animals that presented a body weight loss of more than 25% were closely monitored. To comply with best animal welfare practices, animals that maintained a reduction of more than 25% for the next 24h were sacrificed. However, animals that regained enough weight to be above the -25% threshold after those same 24h were allowed to recover.”

Fig 3b-c – To exclude DAF depletion does not impact viral clearance the authors should extend the time course and show that both cohorts clear the virus at the same rate (ie a later time point showing viral loads is required).

We thank this reviewer for the comment and have now addressed this question by measuring viral titers at days 2, 3, 6, 7 and 9. We observe that WT and *Daf^-/-^* mice have identical lung viral titers at all the measured time points: 2, 3, 6, 7 and 9 days p.i.. At day 9 no viruses were detected (Fig3-A). This extended number of days we have now evaluated enabled us to more strongly conclude that DAF depletion does not impact viral replication and clearance.

Fig 4a – It is unclear as to why the authors dropped the viral dose in this experiment to 500PFU PR8-HK4,6. Moreover, the control of infection of the *Daf^-/-^* with 500 PFU PR8-HK4,6 is missing and needs to be added to this dataset to show at this dose Daf deficiency still improves disease outcomes.

We agree that an additional control was necessary to firmly show that DAF mediated protection operates via C3. In alternative to performing the controls with 500 PFUs, we have now repeated the entire set using 1000 PFUs of PR8-HK4,6 in *Daf^-/-^*, WT, *C3^-/-^* and *C3^-/-^ / Daf^-/-^* mice. As seen in Fig. 4-A, with this higher dose of PR8-HK4,6, the *C3^-/-^* and *C3^-/-^ / Daf^-/-^* mice have similar initial body weight loss, indicating that DAF mediated protection operates via C3. Although only showing data with 1000 PFUs in the manuscript, we reached the same results using two viral doses, which further solidifies our conclusions.

Fig 4b – Assessment of C3a levels in the BAL at a single time point is insufficient to conclude that IAV triggers lower levels of C3a in DAF deficient mice – perhaps levels could have peaked earlier in this cohort. Assessment of C3a levels in the BAL at d0, 3, 6, 10 p.i. would be more convincing evidence for lower complement activation in Daf mice post flu.

We thank this reviewer for the comment and have now addressed this question by measuring C3a levels at days 2, 3, 6 and 7 p.i. as shown in Figure 4-B. We observe that C3a levels are not different at day 2 p.i. but at day 3, 6 and 7 p.i C3a levels are significantly higher in WT rather than *Daf^-/-^*, clearly showing that *Daf^-/-^* have reduced complement activation upon PR8-HK4,6 infection.

Fig 4d-j – Assessing immune cell infiltration into the BAL alone following IAV infection of *Daf^-/-^* vs WT mice is an extremely narrow analysis. It is important that the immune cells infiltrating the lung tissue are also quantified. Moreover, it not clear why the authors chose to normalise the data generated using BAL samples to the WT sample, especially when the variability in the WT group ranged by +/-50%. Absolute numbers/ml of BAL would be more informative.

We acknowledge that further analysis of cell infiltrations in the lung tissue would be of most interest. To address this question, we have analyzed levels of alveolar inflammation, interstitial inflammation and perivascular/peribronchiolar inflammation in H&E stained lung sections of mice infected with PR8-HK4,6, PR8-HK6 and PR8-HK4. According to our pathologist, who performed the analysis blind, the sum of the individual scores for these parameters corresponds to a semi-quantitative measure of cell infiltration in lung parenchyma. Data are represented in Fig. 3-E, 6-G and 8-G for each virus, respectively.

Regarding the flow cytometry data, we understand the concerns raised by the reviewer about showing normalized data for BAL flow cytometry analysis. However, given the variability between batches of antibodies produced in-house, we were not able to pool experiments using absolute values without compromising our data analysis. As an example, we present this figure from two independent experiments performed just two days apart, however using different batches of the in-house α-CD3 antibody. In **A-C** we show the values normalized to WT of the pooled experiments (**A**) and each experiment individually (**B**, **C**), and in **D-F** are represented the corresponding absolute values (**D**: pooled, **E** and **F** individual). In **A**, it is observed that levels of CD8^+^ T cells in *Daf*^-/-^ mice are close to mean value for WT. Conversely, in **D**, despite a similar mean value between WT and *Daf^-/-^* mice, the latter present two populations: one with higher CD8^+^ T cells and one with lower CD8^+^ T cells than the WT. However, in each individual experiment, **E-F**, *Daf*^-/-^ CD8^+^ T cell levels are similar to the mean levels of WT mice. Therefore, the high variability observed in **D** is due to the different batches of antibody used and does not reflect the variation in cell levels. We hope that this clarification is suitable to explain why we present that data normalized.

To avoid such confusion in the data sets, and considering that the main focus of this work is to compare WT vs. *Daf*^-/-^, we have opted to keep the results as percentages of WT in the manuscript. Nevertheless, as we acknowledge the limitations of such analyses and to remove any concern regarding the data, we included absolute values from a representative experiment for each of the viral strains in supplementary data (Fig. S3).

Fig 4k – the assessment of a single cytokine (IFNg) at a single time point (d6) is insufficient to conclude that there is less inflammatory cytokine production in the *Daf^-/-^* mice post influenza. Assessment of a panel of proinflammatory cytokines (including IL-6 which is mentioned but not measured) and chemokines using a bead array type approach will help significantly strengthen this conclusion.

We have followed the advice of this reviewer and performed a multiplex analysis in WT and *Daf^-/-^* mice infected with PR8-HK4,6 1000PFUs, at 3, 6 and 9 days p.i. as presented in Fig. 5 of the manuscript. In the manuscript, we wrote the following text:

Given this result, we thought to further explore the contribution of DAF in modulating inflammatory cues upon viral infection. For that purpose, we assessed a panel of antiviral cytokines at days 3, 6 and 9 p.i. with PR8-HK4,6 in BALs of WT and *Daf^-/-^* mice (Fig. 5). We observe that the content of type I IFN (IFN-α and IFN-β) was not significantly decreased in *Daf*^-/-^ mice at all tested time points (Fig. 5-A, B). As type I IFN play an important role in controlling viral replication (52), this result is in agreement with the similar viral loads observed in WT and *Daf*^-/-^ mice over the course of PR8-HK4,6 infection (Fig. 3-A). Surprisingly, and contrarily to what we had previously shown (Fig. 4-I), this analysis showed no significant difference in levels of IFN-γ were not significantly reduced in *Daf^-/-^* mice when compared to their WT counterparts (Fig. 5-C). Using this system, we observed, a trend of lower IFN-γ levels in *Daf^-/-^* mice at 9 d.p.i. to that observed in WT, that however, did not reach statistical significance, due to the variations within the 5 animals analyzed per condition (Fig. 5-C).

Interleukins (IL) also contribute to excessive inflammation and immunopathology (53). Interestingly, IL-1β, IL-6, IL-10 and IL-12 were not significantly altered upon DAF depletion over the course of infection (Fig. 5-D-G). However, and albeit not significant, we observed an increase in IL-6 content in the BALs of WT mice at 6 d.p.i., that is absent in *Daf*^-/-^ (179.0±193.8 pg/mL vs. 41.2±29.7 pg/mL) (Fig. 5-E). Remarkably, this corresponds to the time point when WT mice present higher lung tissue damage and C3a levels when compared to *Daf^-/-^* mice, as well as higher numbers of neutrophils and macrophages recruited to the lungs (Fig. 3-D, 4-B, E, F). This increase of IL-6 in BALs of WT infected mice could thus explain the increased immune cell recruitment leading to tissue damage and immunopathology.

The potent proinflammatory cytokine tumor necrosis factor (TNF)-α showed an overall trend of being decreased in *Daf*^-/-^ mice, being significantly lower at 9 d.p.i. (34.3±0.5 pg/mL vs. 32.7±0.3 pg/mL) (Fig. 5-H), suggesting that DAF contributes to maintain longer inflammation.

Although no relevant alterations were observed for the granulocyte-macrophage colony stimulating factor (GM-CSF) (Fig. 5-I), *Daf^-/-^* mice had significantly reduced levels of C-C motif chemokine ligand (CCL)2 (or monocyte chemoattractant protein-1, MCP-1) (Fig. 5-J) at 3 d.p.i.. Moreover, *Daf^-/-^* mice did not present the abrupt increase in C-X-C motif chemokine ligand (CXCL)10 observed in WT mice at 6 d.p.i. and had significantly lower levels of this chemokine at 9 d.p.i. (49.7±3.8 pg/mL vs. 63.7±8.7 pg/mL) (Fig. 5-M). CCL2 and CXCL10 being monocyte chemoattractants (54–57), the lower levels of these chemokines in the lungs would explain the reduced numbers of monocytes in PR8-HK4,6 infected *Daf^-/-^* mice when compared to the WT. Interestingly, at 3 d.p.i. *Daf^-/-^* mice also had significantly lower levels of CXCL1. Although we observe reduced neutrophils in *Daf^-/-^* mice at 6 d.p.i. (Fig. 4-E), it is tempting to speculate that this initial reduction in CXCL1 will result in lower neutrophils later in infection. Similarly, at 9 d.p.i., *Daf^-/-^* mice had lower levels of CCL5 (or regulated on activation, normal T cell expressed and secreted, RANTES) than WT mice (77.0±2.0 pg/mL vs. 85.2±8.8 pg/mL) (Fig. 5-K). Although we did not observe significant alterations in CD4^+^ or CD8^+^ T cell recruitment (Fig. 4-G, H), it is tempting to speculate that reduced levels of CCL5 would lead to a faster resolution of the inflammation and faster reduction of T cells at a later time point.

Taken together, these indicate that lower complement activation leads to a reduced antiviral response and recruitment of innate immune cells, such as neutrophils and monocytes.

Fig 6 a- the control of the *Daf^-/-^* alone is missing from this analysis, and a broader time period of assessment for C3 analysis in the BALF is required.

We thank the reviewer for the comment and have included the control *Daf^-/-^* in the data set (Fig. 7-A). Also, we have added the levels of C3a in BALs at day 3 p.i. to increase one time point on the analysis in Fig. 7-B at day 6. Consistent with the modest weight loss observed at this time point, there is no significant difference in C3a levels in BAL of WT and *Daf^-/-^* mice.

For consistency, we also added *Daf^-/-^* control in the survival analysis and acquired C3a data for the PR8-HK4 infection at day 3 p.i. (Fig. 8-A, B). We observe that for PR8-HK4 at day 3 p.i., C3a levels are higher in mice *Daf^-/-^* than WT, which corresponds to similar result in the histological score.

Line 324 – the statement “ that HA-DAF interaction modulates the adaptive immune response “ needs to be tempered or further experimental analysis needs to be performed to support this. This statement is based on assessment of bulk CD4 and CD8 in BALF at a single time point. Either track antigen specific CD4 and CD8 responses over the course of the infection or temper the statement.

We thank and agree with this reviewer’s comment and have substituted the text as “that HA-DAF interaction may be implicated in modulating the adaptive immune response. This hypothesis requires further confirmation through the analysis of a set of markers in BAL over the course of infection”.

Reviewer #2:

The authors thank this reviewer for taking the time to evaluate their manuscript. We have answered each question below, hoping that our answers/data clarifies this reviewer’s concerns. We addressed the questions that were possible, considering the availability of mice to perform experiments, or, with the exception of question 1, we provided alternative means to address the questions raised or as a last resource, explained the reason for not being able to comply with the reviewer’s points. Overall, all the points raised were insightful and we felt that addressing them has made our manuscript scientifically sounder.

1) Determine the C3a concentration over time in WT and DAF-knockout mice after infection with PR8 and X31.

To comply with this reviewer’s request, we have done a complete determination of C3a levels over time in WT and *Daf^-/-^* mice after infection with PR8-HK4,6 that is now included in Figure 4-B. This includes C3a analysis at days 2, 3, 6 and 7 p.i.. Because of shortage of mice, we were not able to perform the same experiments with PR8. Instead, we opted for a thorough analysis using PR8-HK4,6 to be able to answer all the reviewer’s questions regarding the *Daf^-/-^* observed phenotype. This analysis included: a time course of viral titers in the lungs to confirm that viral production and clearance was similar in WT and *Daf^-/-^* mice at days 3, 6, 7 and 9 p.i. (Fig. 3-A); multiplex analyses of several anti-viral cytokines at days, 3, 6 and 9 p.i. (Fig. 5); histological scoring and cell infiltration in lung parenchyma at days 3, 6, and 9 p.i. (Fig. 3-D, E). Finally, we did flow cytometry of neutrophils, monocytes, CD4^+^ T cells and CD8^+^ T cells at days 3, 6 and 9 p.i. (Fig. 4-E-H). We hope this reviewer agrees with our option to really complete the analyses for PR8-HK4,6 that also serves to explain the protection in *Daf^-/-^* mice we observed for viral strains Eng2009 and Cal2009.

2) Determine histology score and immune cell infiltration after PR8 infection in WT and DAF-knockout mice.

We do understand that looking into immune cell infiltration and histological score upon PR8 infection would be really interesting. Due to shortage in *Daf^-/-^* mice we were not able to perform that analysis. However, in order to comply with this request, we are presenting here analyses performed using double knock-out mice *Daf^-/-^ / Cd59^-/-^* because as seen in Fig. 2-E, F, CD59 does not impact IAV infection. Whilst we may not show this in the manuscript, we may certainly show the results to this reviewer with the aim to clarify this question and present two figures below (Figure α and Figure β).

To validate *Daf^-/-^ / Cd59^-/-^* mice as behaving like *Daf^-/-^*, we also included data for PR8-HK4,6. Upon infection with PR8-HK4,6, as observed in infections of *Daf^-/-^* mice, *Daf^-/-^ / Cd59^-/-^* mice had a less severe disease and mortality when compared with their WT counterparts. These mice lost less of their initial bodyweight (12.6% vs. 21.4%) and had reduced mortality than WT mice (50% vs. 100%) (Fig. α-A, B). Analysis of lung viral titers at days 3, and 9 p.i. show that *Daf^-/-^ / Cd59^-/-^* and WT mice have similar lung viral loads during the course of infection. At day 9 p.i. no viruses were detected indicating that mice had cleared the infection (Fig. α-C). Therefore, as observed for *Daf^-/-^* mice, depletion of both DAF and CD59 does not impact PR8-HK4,6 viral replication and clearance. Lastly, we performed a comprehensive and blind histological analysis of lung tissue at days 3, and 9 p.i.. Although there were no significant differences in histological scores nor in cell infiltration in the lung parenchyma between WT and *Daf^-/-^ / Cd59^-/-^* mice at all time points, there is a trend at 9 d.p.i. for *Daf^-/-^ / Cd59^-/-^* mice to have lower values, mimicking the results observed in *Daf^-/-^* (Fig. α-D, E). Taken together, these data confirm that CD59 does not impact PR8-HK4,6 infections in a *Daf^-/-^* background, and therefore *Daf^-/-^ / Cd59^-/-^* have a phenotype equivalent to *Daf^-/-^* mice. *Daf^-/-^ / Cd59^-/-^* mice, thus mimic DAF depletion and may be used to analyze PR8 infection and comply with this reviewer request.

Figure α – *Daf^-/-^ / Cd59^-/-^* mice have a response to PR8-HK4,6 infection comparable to *Daf^-/-^* mice.

**A, B:** Bodyweight loss (**A**) and mortality (**B**) of C57BL/6J WT or *Daf*^-/-^ mice infected with the indicated doses of A/X-31 (PR8-HK4,6). **C**: Lung viral titers of C57BL/6J WT or *Daf*^-/-^ mice infected with 1000 PFU of A/X-31 (PR8HK4,6). Samples collected at 3 and 9 d.p.i.. **D, E**: Histological score (**D**) and cell infiltration in lung parenchyma (**E**) of C57BL/6J WT or *Daf*^-/-^ mice infected with 1000 PFU of PR8HK4,6 was assessed blindly. Results are expressed as mean±sd.

As observed in infection of *Daf^-/-^* mice with PR8, there was no difference in bodyweight loss between *Daf^-/-^ / Cd59^-/-^* and WT mice. Upon sublethal infection, both strains of mice lost comparable body weight, and when infected with a lethal dose of this virus all mice succumbed to the disease at 7 d.p.i. (Fig. β-A, B). Analysis of lung viral titers at days 3 and 9 p.i. showed no difference in lung viral loads between WT and *Daf^-/-^ / Cd59^-/-^* mice over the course of infection indicating that, depletion of DAF does not impact PR8 viral replication and clearance (Fig. β-C). Finally we performed a comprehensive and blind histological analysis of lung tissue at days 3 and 9 p.i.. Histological score and cell infiltration in lung parenchyma was similar in WT and *Daf^-/-^ / Cd59^-/-^* mice at 3 d.p.i. and 9 d.p.i. (Fig. β-D, E). Interestingly, and although not significantly different probably due to the small sample size, we observe a trend of reduced damage and cell infiltration in *Daf^-/-^* mice. Taken together, these results indicate that, as observed in *Daf^-/-^* mice, DAF and CD59 double depletion does not reduce disease severity upon PR8 infection, nor does it impact viral replication and clearance. Interestingly, there is a trend of reduced lung tissue damage, albeit not significant. This data is in accordance with the results obtained in PR8-HK6 infected mice (Fig. 6), and indicate a strong effect of HA-DAF in impacting disease severity, but not viral clearance, replication or tissue damage.

Figure β – *Daf^-/-^ / Cd59^-/-^* are not protected against PR8 infection.

**A, B:** Bodyweight loss (**A**) and mortality (**B**) of C57BL/6J WT or *Daf*^-/-^ mice infected with the indicated doses of PR8. **C**: Lung viral titers of C57BL/6J WT or *Daf*^-/-^ mice infected with 10 PFU of PR8. Samples collected at 3 and 9 d.p.i.. **D, E**: Histological score (**D**) and cell infiltration in lung parenchyma (**E**) of C57BL/6J WT or *Daf*^-/-^ mice infected with 10 PFU of PR8 was assessed blindly. Results are expressed as mean±sd.

We hope that these set of experiments serves to satisfy this reviewer. In the manuscript we have now included a paragraph highlighting the importance of analysis of PR8 infections as future perspectives: “Interestingly, DAF depletion does not ameliorate disease outcome in all IAV infections. We observed that although *Daf^-/-^* mice were protected in Eng, Cal, PR8-HK4,6 and PR8.HK4 infections, levels of mortality were identical in WT and *Daf^-/-^* mice when infected with PR8 and PR8-HK6. These differences could be due to the levels of virulence, PR8 and PR8-HK6 being more virulent than the other strains. However, we have also shown that compared to PR8-HK4,6, infection with PR8-HK6 altered recruitment of adaptive immune cells, and PR8-HK4 of innate immune cells, without changing the levels of C3a in *Daf^-/-^* mice. These results indicate that complement is not the sole recruiter and activator of the immune response, and that a direct or indirect HA-DAF and/or NA-DAF interaction has additional roles to play in immune cell recruitment. Therefore interaction between DAF and different HA and NA could elicit different immune responses. Indeed, although PR8, Eng and Cal are all H1N1 viruses, the corresponding HA and NA proteins are different. Further analysis of PR8, Eng and Cal infections in *Daf^-/-^* mice would allow clarification of the impact of different HA-DAF and NA-DAF interactions.”

3) Test additional “7+1” viruses where you compared additional NA proteins, that either cleave or do not cleave sialic acids from DAF in order to link that observation to an in vivo phenotype.

IAV undergoes rapid evolution, as illustrated by the emergence of resistance to antiviral drugs or reversion of deleterious mutations. Producing viruses with impaired NA activity would add great value to our work and was our original strategy. We have successful produced chimeric PR8 viruses with NAs derived from avian viruses (Fig. S5-A-C). However, when we grew these viruses in 293Ts and amplified them in eggs, they adapted fast to cleaving DAF (Fig. S5-B, E) and therefore our aimed at experimental strategy was not feasible. Hence, upon the impossibility to “bypass” adaptation of the virus to mammalian cells, we addressed this question *in vitro* by comparing C5-b9 deposition in cells transfected with either a fully competent-NA or the catalytic impaired NA-E229A. Given the results, we raise the hypothesis that the NA-mediated DAF cleavage contributes to *in vivo* pathogenicity. We agree that at the moment this hypothesis, however tempting, is merely speculative, and we are planning further experiments to validate it. However, the experiments are not simple, as demonstrated by the fast adaptation of the PR8 NA-H5N2 in A549 cells, and thus this question will be addressed in a future study. In the manuscript, we now address this as:

To validate this hypothesis, we aimed at engineering recombinant mutant viruses composed of seven PR8 segments (segments 1-5, 7-8) and expressing segment 6 from viruses that do not cleave DAF. If indeed our hypothesis is correct, the absence of DAF cleavage would supress the protective effect observed in *Daf^-/-^* mice. Supported by data in Fig. 10-I, we selected segment 6 from H5N2 and H5N8 virus to produce RG 7+1 PR8 reassortant viruses (Fig. S5-A). After transfecting HEK293T cells with the eight plasmids to produce the P0 viruses, we observed that PR8 NA-H5N2 did not cleave DAF, as expected (Fig. S5-B). However, PR8 NA-H5N8 viruses acquired the capacity to cleave DAF (Fig. S5-B). For this reason, even though we amplified both viruses in eggs to avoid additional adaptation to host environment (Fig. S5-C), we excluded the PR8 NA-H5N8 from further experiments as they had acquired the ability to cleave DAF. The rescued PR8 NA-H5N2 in eggs (Fig. S5-C), was then tested for its ability to infect and replicate in A549 cells. One-step infection at high MOI resulted in effective replication, though PR8 NA-H5N2 grew to a significantly lower extent than PR8 (Fig. S5-D). Remarkably, when A549 cells were infected, PR8 NA-H5N2 cleaved DAF (Fig. S5-E). These results strongly suggest that IAVs containing NAs from avian origin rapidly adapt to cleave DAF α2,6-linked sialic acid, and could not be used to assess the link between DAF cleavage *in vitro* and pathogenicity upon mouse infection.

Hence, to assess the effect of DAF sialic acid cleavage in complement activation and to overcome these mechanisms of fast adaptation, we opted for a completely *in vitro* approach. We introduced the mutation E229A in PR8 segment 6, which pronouncedly decreases NA enzymatic activity, while still sustaining a low level of viral replication (72). We then produced lentiviral vectors to deliver WT or E229A versions of PR8 NA fused to GFP, and confirmed that NA-E229A had impaired DAF cleavage (Fig. 10-J).

4) The depiction of C3 in the model figure is not fully supported by the data. How is HA expression linked to complement activation? Same with sialic acid cleavage of DAF by NA. Because different doses of virus were used, it is difficult to compare C3a levels between PR8, X31, and the H3 and N2 only viruses. Without this information, you cannot conclude that sialic acid cleavage of DAF affects activity in vivo and thus C3a concentration.

We acknowledge that the model needs further validation and have highlighted where we lack data (Fig. 11). Figure legend has been corrected as follows: “At steady state, DAF accelerates the decay of C3 convertases, inhibiting the formation of C3a and C3b and subsequent complement activation. Upon IAV infection, the cell will produce viral proteins, and in particular HA (in blue) and NA (in orange). NA is a potent sialidase that will remove the sialic acid content of DAF both in the cytoplasm and at the surface. This processing of DAF by NA may lead to DAF loss/alteration of function. Desialylation of DAF would then impair the inhibition of C3 convertases leading to overactivation of the complement pathway. In parallel, the presence of HA at the cell surface may exacerbate complement activation that could then not be controlled by a desialylated DAF. The activation of complement pathway via HA, combined with the lack of inhibition due to NA sialidase activity would induce complement overactivation, leading to C3a release. Moreover, the presence of HA at the cell membrane and recognition of desialylated surface proteins by PRR would induce an exacerbated immune response (red asterisks). Consequently, an excess of innate immune cell recruitment and inflammation would promote tissue damage and ultimately immunopathology, worsening disease outcome. (Black arrows indicate confirmed events whereas grey arrows indicate speculative events needing further validation. Red arrows indicate events that will worsen disease outcome.)

**Part III – Minor Issues: Editorial and Data Presentation Modifications**
Please use this section for editorial suggestions as well as relatively minor modifications of existing data that would enhance clarity.

Reviewer #1: Fig 4d- scale has a typo

We thank the reviewer for this comment. Figure 4 has been corrected.

Table S1 – Some representative histological profiles showing examples of disease pathology would be helpful.

Representative images of histological samples of each infection have been added in Fig. S1.

Fig 5-7 – the results generated with the hybrid PR8-HK6 or PR8-HK4 are confounded by the increased virulence of these strains compared to PR8-HK6,4 which sees every set of experiments performed with different doses of viruses. The authors should acknowledge this caveat. Moreover, this section is difficult to understand and I recommend the authors try simplifying the message.

We do understand the concern of this reviewer and have acknowledged the caveat, adding the following sentence to the text: “Furthermore, given the variability in virulence between viral strains, mice were infected with different PFUs of each virus in order to obtain similar mortality and weight loss of WT mice, and thus compare the impact of DAF depletion in these experiments. “

Reviewer #2: Minor edits:

1) Normalizing the flow data to WT may exacerbate small differences. It is better to present the actual cell numbers.

We have acknowledged this reviewer’s concerns, which was also raised by the other reviewer and hence we copy the same explanation here. Although batches of antibodies do not allow us to pool absolute values, and so we will maintain percentages in the main figures, absolute values of representative experiments are now shown in Fig. S2.

We understand the concerns raised by the reviewer about showing normalized data for BAL flow cytometry analysis. However, given the variability between batches of antibodies produced in-house, we were not able to pool experiments using absolute values without compromising our data analysis. As an example, we present this figure from two independent experiments performed just two days apart, however using different batches of the in-house α-CD3 antibody. In **A-C** we show the values normalized to WT of the pooled experiments (**A**) and each experiment individually (**B**, **C**), and in **D-F** are represented the corresponding absolute values (**D**: pooled, **E** and **F** individual). In **A**, it is observed that levels of CD8^+^ T cells in *Daf*^-/-^ mice are close to mean value for WT. Conversely, in **D**, despite a similar mean value between WT and *Daf^-/-^* mice, the latter presents two populations: one with higher CD8^+^ T cells and one with lower CD8^+^ T cells than the WT. However, in each individual experiment, **E-F**, *Daf*^-/-^ CD8^+^ T cell levels are similar to the mean levels of WT mice. Therefore, the high variability observed in **D** is due to the different batches of antibody used and does not reflect the variation in cell levels.

To avoid such confusion in the data sets, and considering that the main focus of this work is to compare WT vs. *Daf*^-/-^, we have opted to keep the results as percentages of WT in the manuscript. Nevertheless, as we acknowledge the limitations of such analyses and to remove any concern regarding the data, we included absolute values from a representative experiment for each of the viral strains in supplementary data (Fig. S3).

2) Is A/England/159/2009 also a pdmH1N1 virus and if so, why is the phenotype different from A/Cal/7/2009?

We thank this reviewer to raise this point, which might need clarification. Despite both being circulating viruses from 2009 pandemic, these are different isolates. It has been reported that variants with different virulence co-circulated during the pandemic, and alterations in a single aminoacid were sufficient to alter virulence in a manuscript published by Amelia Nieto where we collaborated (Nieto A *et al*., 2019, Mutation S110L of H1N1 influenza virus hemagglutinin: A potent determinant of attenuation in the mouse model. Front. Immunol 10, Article 132, <https://doi.org/10.3389/fimmu.2019.00132>). Therefore, it is not surprising that the mentioned viruses present different phenotypes.

4) Consider legend location for Fig 3 between A-B and D-F (horizontal).

This reviewer’s comment has been taken into consideration, and figure was corrected (Fig. 3).

5) Fig legend 2S does not appear to have a F and G section.

We thank the reviewer for this comment. The figure legend (now Fig. S4) has been corrected as follows: “**F**, **G**: Cell death of primary lung cells derived from WT or *Daf*^-/-^ mice infected or mock-infected with PR8, PR8-HK4,6, PR8-HK4 or PR8-HK6 and treated with serum **(F)** or heat-inactivated control **(G)**. Results are expressed as mean±sd from 3 replicates from 2 independent experiments. Statistical analysis detailed in materials and methods”.
